# Concentrated Foods

**Published:** 1891-04

**Authors:** 


### Concentrated Foods.

Among the products that properly have their sale through druggists are the concentrated foods which the intelligent physician of to-day finds an indispensable adjuvant to medicinal means of sustaining nutrition in diseases attended with enfeeblement of the digestive and assimilative functions.

What may be termed hypernutrition is now an established and important method of treatment in all wasting diseases, and notably in the great devastator of human life, pulmonary consumption.

The role of concentrated foods is not limited, however, to the invalid alone. The problem of affording armies, large forces of laborers, or the inmates of large charitable institutions an available concentrated nutritious diet is one of the greatest importance.

Dietetics, or the selection of proper alimentation representing the highest nutritive power and requiring the least effort of the digestive functions, is an art in which progress is of national interest.

One need only allude, as illustrations of the application of such foods, to the great army of dyspeptics whose views of life are embittered by their inability to digest ordinary foods, and to the alarming prevalence during the hot months of infantile disorders dependent largely on improper or irritating diet. —*Druggists' Bulletin.*

